# Social Workers’ Perspectives on the Climate of Trauma-Informed and Non-Trauma-Informed Schools

**DOI:** 10.1007/s40653-026-00857-8

**Published:** 2026-03-17

**Authors:** Kate R. Watson, Ron Avi Astor, Gordon P. Capp, Rami Benbenishty

**Affiliations:** 1 Luskin School of Public Affairs, University of California, Los Angeles, USA; 2 School of Education and Information Studies, University of California, Los Angeles, USA; 3 Department of Social Work, California State University, Fullerton, USA; 4 The Paul Baerwald School of Social Work and Social Welfare, The Hebrew University of Jerusalem, Jerusalem, Israel; 5 Education and Social Sciences School, Universidad Andrés Bello, Santiago, Chile

**Keywords:** Trauma informed, School climate, School social work, School safety, Trauma

## Abstract

This study explored school climate from the perspective of social workers, documenting their perceptions of trauma-informed culture and climate in U.S. schools. Using logistic regression, we analyzed survey data from 538 school social workers to investigate whether their experiences of school climate characteristics associated with trauma-informed approaches—physical and psychological safety; a focus on strengths and equity; and supportive relationships—were associated with their identification of a school as trauma informed. We found that social workers perceived clear climate differences between schools they identified as trauma informed and those they did not. ​Schools that were perceived as safer; with a strengths-based, equitable focus; and providing trusting, empowering, and collaborative relationships were more likely to be identified as trauma informed. These differences do not relate to any specific curriculum or program and are commitments any school could make. With adequate time and influence, school social workers can support and encourage positive shifts in trauma-informed climate as part of their everyday efforts toward building and sustaining positive school climate.

The importance of school staff in creating a positive school climate has long been recognized in the school safety practice and implementation literature (Thapa et al., 2013; Voight & Nation, 2016). However, few studies have examined how school social workers perceive or understand their school settings. In part, this is because school staff and their views have rarely been considered in empirical research of school safety or school climate (Berkowitz et al., 2017; Capp et al., 2020; Wang & Degol, 2016). With increasing recognition that non-teaching school staff are missing from research literature (Capp et al., 2020), school climate is beginning to be empirically explored from the perspective of other school staff members. According to the latest practice models (Tan & SSWAA, 2024), support of a positive school climate has been identified as an essential part of the school social worker role. As such, social workers’ views of the climate of schools they serve are essential.

Reports by school social workers are particularly salient to the study of trauma-informed climate because of their graduate-level training in behavioral and mental health and their role supporting the well-being of children and families in schools, including those who have experienced trauma (Watson et al., 2022; Kelly et al., 2015, 2021; Phillippo et al., 2017). The aim of this study was to determine if school social workers reported culture and climate experiences—that is, the presence of trauma resources, school safety, a strengths-based, equitable focus, and high-quality relationships—that differed between schools they identified as trauma informed and other schools. Although trauma training and trauma-focused evidence-based practices (EBPs) have become quite popular in schools over the past decade, it is unclear whether training or the availability of clinical trauma interventions relate to any differences in a school’s climate.

## Trauma-Informed Practices in U.S. Schools

In recent years, trauma-informed practices have gained significant traction within the U.S. K-12 public education system. The incorporation of trauma-informed practices in schools relates to the recognition that most youth report experiencing violence or victimization before age 18 and that such experiences can negatively impact children’s ability to focus and learn (Perfect et al., 2016; Turner et al., 2010). Trauma-informed schools attempt to avoid traumatizing students and families by ensuring that all staff understand and can recognize trauma symptoms, and that school policies, practices, and procedures actively avoid perpetuating harm (Substance Abuse and Mental Health Services Administration, 2014). A notable increase in state-level adoption of trauma-informed approaches in schools was observed in 2017, with 29 state education agencies submitting plans to the U.S. Department of Education that incorporated trauma-informed strategies. This trend continued to accelerate, with 45 state education agencies publicizing plans to develop trauma-informed schools by 2019 (Simon et al., 2020). Despite this rapid expansion, empirical evidence supporting specific components and processes needed for a whole-school trauma-informed approach remains limited (Avery et al., 2021; Gherardi et al., 2020; Maynard et al., 2019; Thomas et al., 2019). To address this gap, researchers have called for a comprehensive examination of current practices and conceptualizations of trauma-informed approaches in schools (Maynard et al., 2019).

## The Role of Social Workers in Providing Trauma-Informed Care in Schools

In many U.S. schools, social workers serve as frontline behavioral and mental health providers. They make school–community linkages, may implement social emotional learning programs (Kelly et al., 2015, 2021), and their therapeutic caseloads typically include children with trauma histories and a range of other behavioral and educational concerns (Watson et al., 2022; Phillippo et al., 2017). In addition to providing direct services to students and their families, the latest practice model calls for school social workers to work across the spectrum, from micro-level, interpersonal interventions through mezzo-level school climate and organizational supports, to macro-level communitywide and global policy and advocacy efforts (Tan & SSWAA, 2024). Since climate is believed to be an essential component of the school social work role, social workers’ views of the climate of schools they serve are essential. Social workers are also well-suited to drive change efforts to introduce and sustain trauma-informed climate, given current practice models and their trauma-related expertise (Dombo & Sabatino, 2019; Tan & SSWAA, 2024).

## A Brief History, Conceptual Model, and Measurement of School Climate

School climate was identified as a key factor in students’ experiences and learning more than 100 years ago (Cohen et al., 2009). Empirical school climate research began in the 1950s, born out of industrial/organizational research and the recognition that differences between schools seemed to relate to differential student outcomes (Cohen et al., 2009; Thapa et al., 2013). There are many different definitions and categorizations of school climate, but four dimensions are commonly associated with it: (1) physical and social-emotional safety; (2) the teaching and learning environment, including instructional quality, administration, and professional development opportunities; (3) relationship quality among school staff, between staff and students, and with the broader community; and (4) environmental and structural factors, including physical and operational characteristics of the school (e.g., school size and space adequacy, and curricular and extracurricular offerings; Cohen et al., 2009; Thapa et al., 2013; Wang & Degol, 2016). Benbenishty and Astor (2024) reimagined school climate as three planes that intersect to create a cube: the social–relational plane, which includes the quality of school-based relationships; the academic plane, which encompasses the school’s teaching and learning structures and environment; and the organizational plane, which captures school leadership and decision making, and practices and policies that affect school operations. Each plane of the cube interacts with the other planes in every aspect of staff members’ and students’ experiences and the school’s operations.

Wang and Degol (2016) found that 90% of school climate research utilized self-report surveys. Surveys are useful because they allow multiple dimensions of climate to be captured simultaneously and they are inexpensive to administer (Wang & Degol, 2016). Multidimensional models like school climate require multifaceted assessment and the voices of multiple constituents; however, most school climate research has focused on student experiences to the exclusion of staff members’ perceptions, and fewer than 1 in 5 studies solicited multiple perspectives (Berkowitz et al., 2017; Wang & Degol, 2016). Given that personal characteristics such as the race, gender, and role of a respondent are significant factors in school climate perceptions (Thapa et al., 2013), it is essential that multiple perspectives are solicited. Also, since staff members are tasked with creating a positive school climate for students, researchers are coming to agree that they too must experience the benefits of a positive climate (Bloom, 1995; Capp et al., 2020; Yoder et al., 2018). Where they do not, the school climate suffers overall.

## The Need to Reintegrate Organizational Culture and Climate Theory in the Study of School Climate and Trauma-Informed Approaches

While often used interchangeably, organizational culture and climate are not synonymous. Organizational culture is the ethos of an organization, its values, rationale for being, and behavioral expectations. Organizational climate is the shared perceptions of policies, processes, and routines among all members of an organization, or the combined psychological impact of the organization (Glisson, 2015; Ostroff et al., 2013; Schneider et al., 2013). Changes in organizational practices, policies, and procedures are theorized to lead to a changed organizational climate; however, little research has tested these mechanisms (Ostroff et al., 2013). A commitment to becoming trauma informed is intended to shift organizational culture (Bloom & Yanosy Sreedhar, 2008; Harris & Fallot, 2001) and result in changed climate perceptions among staff and service recipients.

The purpose of this study was to determine whether social workers’ reports of organizational culture and climate were different between trauma-informed and non-trauma-informed schools. We also wanted to determine whether certain characteristics of trauma-informed culture and climate were associated with whether a school would be identified as trauma informed. The research questions for this study were: To what extent did school social workers report culture and climate differences between schools they identified as trauma informed and those that were not? Could certain characteristics of trauma-informed culture and climate indicate whether a school was identified as trauma informed?

## Methods

### Population and Study Samples

**Fig. 1:**
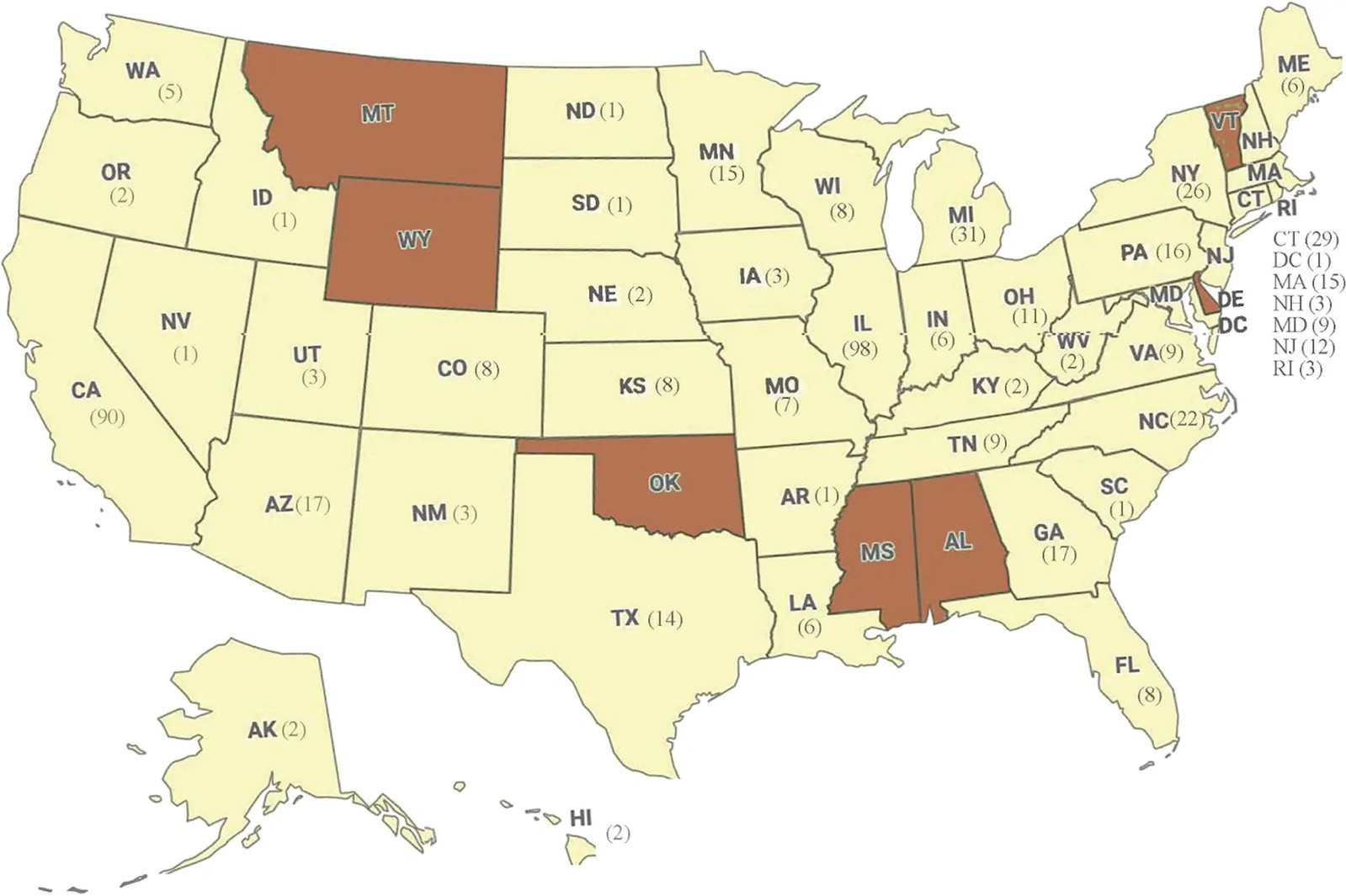
States where participants practice

This study was based in part on the first author’s dissertation research (Watson, 2024) Data presented here are a subset of a larger data collection among a population of school social workers across the United States. We partnered with several professional associations, including the National Association of Social Workers, School Social Work Association of America, School Social Work Network, who shared a link to the anonymous online survey with their members. Respondents were school-based social workers, social work supervisors, or district heads of social work services. The sample (*N* = 538) was a convenience sample representing 43 U.S. states and the District of Columbia (see Figure 1), with the highest concentration from Illinois (18.3%), California (16.8%), Michigan (5.8%), and Connecticut (5.4%). Detailed demographics of the sample are presented elsewhere (Watson, 2024; Watson et al., 2024). Most participants self-identified as female (90.7%), 7.2% as male, 0.4% as gender non-conforming, and others chose not to disclose. The sample primarily identified as White/Caucasian (72.7%), 11.7% as Hispanic/Latinx, 7.4% as Black/African American, 4.3% as Multiracial, 0.7% as Asian American, 0.2% as Hawaii Native/Pacific Islander, and 0.2% as Native American/Alaska Native, and others chose not to disclose. Practicing school social workers were the primary respondents (89.2%), 1.5% identified as district social work supervisors, 0.9% were heads of social work services in a district, 0.6% were school-based social work contractors, and those remaining held other school-based positions or chose not to disclose. Participants’ years of experience ranged from less than 1 (4.3%) to more than 20 (23.7%), with a mean of more than 10 years of social work experience. Participants served a variety of school types, including preschool (17.1%), elementary (57.6%), middle or junior high (48.3%), high school (44.8%), and alternative schools (12.8%). Many participants reported serving multiple schools simultaneously.

School characteristics reported by participants have been published (Watson, 2024; Watson et al., 2024). Participants worked in suburban (43.3%), urban (38.1%), and rural (18.2%) districts across the United States. Overall, participants reported working in high-need schools, where a mean of 63.8% of students received free/reduced lunch. On average, 55.7% of students were from historically marginalized populations; 19.5% of district students reportedly drop out, and 55.5% enter college.

Note: States marked as yellow were represented among participants. The bracketed number indicates the number of participants from that state. Brown states had zero participants.

## Instrument and Ethics

Survey questions were developed by the research team to understand the needs of school staff, students, and families during the 2021–2022 school year, and the relationship between those needs and current models for trauma-informed schools. The instrument included both closed- and open-ended questions about schools’ programs and policies and solicited school social workers’ views about the trauma-informed culture and climate of their school environment. Self-report data is utilized in 90% of school climate research (Wang & Degol, 2016). This research was found to be exempt from institutional review board approval (IRB#22–000343), and partner organizations completed their own internal ethics review processes.

While there are several systems-level measures to assess values, belief systems, and activities of a trauma-informed environment (see Champine et al., 2019), few published instruments assess trauma-informed climate. Based on a prior review of common components of a trauma-informed approach (Watson & Astor, 2025), we developed a new tool to assess trauma-informed culture and climate. The instrument comprised four modules: (a) trauma commitment and training (i.e., trauma-informed culture); (b) physical and psychological safety; (c) strengths-based, equitable focus; and (d) trust-based, collaborative, and empowering relationships. The latter three modules assess dimensions of trauma-informed climate. Survey items are presented in Table 1. Experts in survey design and trauma-informed organizations were consulted during instrument development to assess content and construct validity. Subsequently, cognitive interviews were conducted to determine whether survey questions were clear and yielded responses across the Likert scale. Cronbach’s alpha was used to assess internal reliability of each module. This study served as a pilot for the instrument. A variation specific to volunteer organizations is also being tested.

**Table 1:** School social worker reports of trauma-informed culture and climate in schools identified as trauma informed (TI) and not (NTI)

Culture and climate variables	TI (*n* = 154)			NTI (*n* = 378)				
	M	SD		M	SD	t _(df)_	*p*	Cohen’s d
Staff training and commitment (α = 0.800)								
All staff in this school receive trauma training.	3.37	1.20		1.82	0.92	15.96_(529)_	< 0.001	1.526
Leadership are committed to being trauma informed.	3.50	1.01		2.28	0.93	13.37_(530)_	< 0.001	1.279
Staff understand the importance of and prioritize self-care.	3.29	0.92		2.59	0.76	9.03_(526)_	< 0.001	0.864
This school adapts policies and procedures to promote emotional, physical, and psychological well-being.	3.34	1.03		2.57	0.90	8.59_(530)_	< 0.001	0.821
I feel confident recognizing the signs and symptoms of trauma in myself and others.	4.49	0.67		4.17	0.87	4.10 _(527)_	< 0.001	0.392
Training and commitment index	3.60	0.70		2.68	0.61	14.99_(523)_	< 0.001	1.437
Safety (α = 0.906)								
It is safe to discuss difficult topics.	3.38	1.14		2.77	1.09	5.75_(528)_	< 0.001	0.551
Staff feel a sense of belonging and connection to this school and staff.	3.40	1.02		2.90	1.05	5.00_(527)_	< 0.001	0.479
When students feel overwhelmed, there is a safe space for them to calm down.	3.92	1.08		3.41	1.12	4.86_(529)_	< 0.001	0.465
There is someone with whom staff can express difficult emotions.	3.53	1.20		2.98	1.21	4.69_(530)_	< 0.001	0.448
The school environment is free from all types of discrimination.	3.13	1.12		2.68	1.08	4.30_(526)_	< 0.001	0.412
It is safe to make suggestions, without fear of consequences.	3.50	1.18		3.06	1.22	3.83_(529)_	< 0.001	0.366
The school makes appropriate safety accommodations, when needed.	3.74	0.96		3.38	1.03	3.73_(530)_	< 0.001	0.356
It is safe to take risks and make mistakes.	3.24	1.13		2.85	1.14	3.63_(528)_	< 0.001	0.348
Students feel a sense of belonging and connection to this school and staff.	3.40	0.88		3.13	0.88	3.21_(530)_	0.001	0.306
The physical environment at my school feels safe.	3.84	0.92		3.56	1.06	2.88_(530)_	0.004	0.275
The school environment is free from verbal, physical, or sexual violence.	3.41	1.29		3.13	1.24	2.25_(529)_	0.025	0.215
Safety index	3.49	0.78		3.08	0.78	5.43_(517)_	< 0.001	0.526
Strengths-based, equitable focus (α = 0.950)								
Staff are comfortable addressing racism and discrimination.	3.10	1.16		2.41	1.03	6.72_(528)_	< 0.001	0.643
School leadership addresses racism and discrimination appropriately.	3.27	1.23		2.56	1.09	6.55_(526)_	< 0.001	0.628
All races, ethnicities, religions, and cultural traditions and backgrounds are valued.	3.78	1.08		3.19	1.07	5.77_(529)_	< 0.001	0.552
All genders are respected and affirmed.	3.71	1.00		3.14	1.08	5.70_(527)_	< 0.001	0.546
Staff strengths are acknowledged and valued.	3.39	1.03		2.86	0.99	5.52_(529)_	< 0.001	0.528
Everyone is welcomed and supported.	3.58	1.13		3.09	1.02	4.89_(528)_	< 0.001	0.467
Students are treated fairly and equitably.	3.55	1.00		3.11	1.00	4.64_(528)_	< 0.001	0.444
At this school, staff matter.	3.63	1.03		3.18	1.04	4.54_(529)_	< 0.001	0.434
Staff are treated fairly and equitably.	3.43	1.14		2.95	1.06	4.65_(528)_	< 0.001	0.444
Student strengths are acknowledged and valued.	3.66	0.92		3.27	0.95	4.39_(529)_	< 0.001	0.420
Staff interactions are consistently respectful.	3.59	0.94		3.19	0.98	4.30_(528)_	< 0.001	0.411
At this school, students matter.	4.14	0.87		3.78	0.92	4.16_(529)_	< 0.001	0.397
Strengths-based, equitable focus index	3.57	0.85		3.06	0.80	6.55_(518)_	< 0.001	0.630
Trust-based, collaborative, and empowering relationships (α = 0.944)								
Staff have a voice in school decision-making.	3.02	0.99		2.42	1.00	6.29_(523)_	< 0.001	0.605
Staff feel empowered to make decisions related to their work and priorities.	3.03	0.92		2.50	0.96	5.77_(526)_	< 0.001	0.553
Students feel empowered to make decisions related to their work and priorities.	2.95	0.91		2.44	0.91	5.83_(524)_	< 0.001	0.559
Students and families have a voice in school decision-making.	2.92	0.99		2.43	0.90	5.82_(524)_	< 0.001	0.523
Staff have trust-based relationships with supervisors and colleagues.	3.17	0.96		2.67	0.97	5.42_(528)_	< 0.001	0.521
Leadership encourages collaboration across roles.	3.40	1.06		2.82	1.17	5.25_(527)_	< 0.001	0.504
Across the school organization, people collaborate effectively.	3.21	1.00		2.72	0.98	5.19_(525)_	< 0.001	0.499
Leadership makes decisions transparently.	2.93	1.12		2.45	1.09	4.58_(525)_	< 0.001	0.439
Staff communicate expectations clearly and consistently to students.	3.46	0.94		3.07	0.96	4.34_(526)_	< 0.001	0.418
Leadership communicates expectations clearly and consistently to staff.	3.14	1.11		2.69	1.12	4.30_(528)_	< 0.001	0.412
Students have trust-based relationships with staff.	3.64	0.80		3.30	0.90	4.08_(526)_	< 0.001	0.392
Staff and administrators role model appropriate behavior.	3.58	0.92		3.22	0.99	3.89_(527)_	< 0.001	0.374
Staff manage their emotions appropriately.	3.27	0.88		3.03	0.94	2.68_(526)_	0.008	0.258
Trust-based, collaborative, empowering relationships index	3.20	0.76		2.75	0.75	6.26_(515)_	< 0.001	0.608

## Measures

### Personal Characteristics

Respondents were asked to report on their role (i.e., school social worker, district supervisor, head of services in a district, school-based contractor, or other), their U.S. state of practice, the types of schools they support (e.g., preschool, elementary, etc.), and the number of years they practiced as a school social worker. They were also asked to report their gender identity and race/ethnicity.

### School Characteristics

Participants were asked to report on characteristics of their school district, including their estimates for the percentage of students eligible for free/reduced lunch, from historically marginalized populations, who drop out, and who enter college. They also reported on the school’s community setting (i.e., urban, suburban, rural).

### Dependent Variable: Report of Whether the School is Trauma-Informed or Not

Participants were asked to choose one school with which they work and to indicate whether that school was considered a trauma-informed setting. Because there are no certifications for trauma-informed schools and limited consensus regarding the characteristics of a trauma-informed school, this study focused on perceptions of school social workers as to whether or not a school was trauma informed, given their relevant expertise.

### Independent Variables: Trauma-Informed Culture and Climate Scales

Three scales assessed trauma-informed climate and one assessed trauma-informed culture. Answers were on a 5-point Likert scale where 1 = Not at all, 2 = To a small extent, 3 = To a moderate extent, 4 = To a large extent, and 5 = To a very large extent. See Table 1 for a complete list of questions.

**Trauma Training and Commitment Scale**. The trauma training and commitment scale, which assessed school culture related to trauma-informed care, comprised five questions (α = 0.80) about the school’s commitment to becoming trauma-informed and providing staff with relevant training. Sample questions included, “All staff in this school receive trauma training” and “This school adapts policies and procedures to promote emotional, physical, and psychological well-being.”

**Physical and Psychological Safety Scale**. The physical and psychological safety scale assessed one dimension of trauma-informed climate. It consisted of 11 questions (α = 0.91), including, “When students feel overwhelmed, there is a safe space for them to calm down” and “The physical environment at my school feels safe.”

**Strengths-Based**,** Equitable Focus Scale**. The strengths-based, equitable focus scale assessed a second dimension of trauma-informed climate. It comprised 12 questions (α = 0.95), including “Staff are comfortable addressing racism and discrimination” and “Students are treated fairly and equitably.”

**Trust-Based**,** Collaborative**,** and Empowering Relationships Scale**. The trust-based, collaborative, and empowering relationships scale assessed a third dimension of trauma-informed climate. It consisted of 13 questions (α = 0.94), including “Staff feel empowered to make decisions related to their work and priorities” and “Across the school organization, people collaborate effectively.”

### Analysis

Preliminary analyses consisted of independent samples t-tests to compare school social workers’ experiences of trauma-informed culture and climate in schools they identified as trauma informed as compared to other schools. Hierarchical logistic regression was then utilized to compare indices of school culture and climate with a single dichotomous outcome variable indicating whether schools were considered trauma informed. Respondent and school characteristics were controlled for in Step 1.

## Results

Bivariate analyses showed an association between social worker-identified school type (i.e., trauma informed or not) and culture and climate indices (i.e., commitment to trauma; psychological and physical safety; strengths-based, equitable focus; and positive school-based relationships). For example, participants who reported working in trauma-informed schools (*M* = 3.57, *SD* = 0.85) compared to the participants in non-trauma-informed schools (*M* = 3.06, *SD* = 0.80) indicated their schools were significantly more likely to demonstrate a strengths-based, equitable focus, *t*(518) = 6.55, *p* < .001. Participants in trauma-informed schools reported more positive culture/climate on every index *and* variable assessed. Effect sizes, as measured by Cohen’s *d*, ranged from *d* = 0.526 for the safety index to *d* = 0.630 for the strengths-based, equity focus index, indicating that school type had a medium effect on climate indices. The effect size of the culture index (i.e., a school’s commitment to trauma) was *d* = 1.437, indicating a very large effect. Full results are presented in Table I.

Multiple hierarchical logistic regressions were performed with social workers’ reports of whether a school was trauma informed serving as the dependent variable. Participant demographics (e.g., race, gender) and school characteristics (e.g., urbanicity, U.S. region, student demographics, and achievement levels) were entered in Step (1). One climate scale was then added in Step (2). None of the models that included solely participant and school characteristics was significant. Each model incorporating an individual climate scale was statistically significant, suggesting that differences in school climate characteristics did exist between schools that social workers identified as trauma informed and those they did not. The safety index was significant (*X*^*2*^(18) = 68.621, *p* < .001) with good model fit indicated by non-significant Hosmer-Lemeshow test result and Nagelkerke R-squared of 0.291. Schools perceived to be safer by social workers were 3.7 times more likely to be identified as trauma informed (*p* < .001). The strengths and equity index model was significant (*X*^*2*^(18) = 77.663, *p* < .001) with a good model fit and Nagelkerke R-squared of 0.316. Schools perceived by social workers to have a greater focus on strengths and equity were more than 3.8 times as likely to be identified as trauma informed (*p* < .001). The model incorporating the relationships index was significant (*X*^*2*^(18) = 70.908, *p* < .001) with good model fit and Nagelkerke R-squared of 0.294. Schools in which social workers reported having higher-quality relationships were 3.8 times more likely to be identified as trauma informed (*p* < .001). The personal and school characteristics found to be significant in the above models were respondents’ reported years as a school social worker and the percentage of historically marginalized students among the student population.

A final logistic regression incorporated all culture and climate scales simultaneously (see Table 2). The goal was to determine the individual and combined effects of each of the culture and climate indices. Participant and school characteristics found significant in prior analyses—i.e., years of experience as a social worker and historically marginalized student population—were included in the final model. The model incorporating all culture and climate indices was statistically significant (*X*^*2*^(6) = 170.329, *p* < .001). This model had a non-significant Hosmer-Lemeshow test result and Nagelkerke R-squared of 0.425. The inclusive model correctly classified trauma-informed schools 76.4% of the time and correctly classified all schools 77.4% of the time. In this final model, the only index found to be significant was training and commitment (*p* < .001). Schools with a commitment to trauma and trauma training were 8.3 times more likely to be identified by school social workers as trauma informed. Although, in earlier regressions, all climate indices were associated with whether a school would be identified as trauma informed, clearly the most important contributor was the presence of a commitment to trauma and related training. These findings may relate in part to the high correlation between the culture and climate indices. For example, the safety, strengths and equity, and relationship indices all have moderate correlations (0.516, 0.568, and 0.545 respectively) to the trauma training and commitment index and high correlations of between 0.818 and 0.880 among each other. We assessed indices’ variance inflation factors (VIF) to determine the extent to which multicollinearity was inflating the values of regression coefficients. VIF was in the moderate range (1.48–5.40) across all indices. Given that only the final regression model included multiple indices, we believe the presented analysis was appropriate.

**Table 2:** *Logistic Regression Predicting Trauma-Informed Schools by Culture and Climate Indices*.

Constant	b		SE		OR		95% CI
	-7.030		0.856		0.001		
Years as a Social Worker	− 0.149		0.069		0.861*		0.753, 0.985
Historically Marginalized Student Population	0.009		0.004		1.009*		1.001, 1.018
Training & Commitment Index	2.111		0.236		8.253***		5.193, 13.116
Collaborative, Empowering Relationships Index	0.215		0.352		1.240		0.622, 2.471
Safety Index	− 0.200		0.300		0.819		0.455, 1.473
Strengths-based, Equity Index	− 0.067		0.346		0.935		0.475, 1.841
Model χ^2^ (df) =	170.329 (6)***						
Nagelkerke R^2^ =	0.425						
n =	477						

*** p < .001 ** p < .01 * p < .05

Respondent and school characteristics found to be significant in prior models remained significant in the final model. Social worker respondents with more experience were less likely to report working in a trauma-informed school, with an odds ratio of 0.861 (95% CI [0.753, 0.985]). Schools with a higher population of students from historically marginalized backgrounds were more likely to be identified as trauma informed, with an odds ratio of 1.009 (95% CI [1.001, 1.018]).

## Discussion

This study sought to identify whether social workers experienced differences in organizational culture and climate between schools they reported to be trauma informed and those they did not. Across all four of our culture and climate indices, they did. Social workers’ perceptions of a school’s trauma commitment and training; physical and psychological safety; strengths-based, whole person, and equitable focus; and the presence of positive, empowering, and collaborative relationships were all positively associated with whether social workers indicated the school was trauma informed. In a field where much uncertainty remains as to exactly what characteristics are required to call a school trauma informed and what outcomes relate to trauma-informed schools, these are important findings.

### Climate Differences Between Schools Identified as Trauma Informed and Not

School social workers indicated that all four elements of trauma-informed culture and climate we assessed—trauma commitment and training; physical and psychological safety; strengths-based, equitable focus; and positive, empowering, and collaborative relationships—were individually associated with whether a school was identified as trauma informed. These associations went above and beyond any differences in respondent or school characteristics. Schools that social workers deemed safer were 3.7 times more likely to be identified as trauma informed. Schools with a strengths-based, equitable focus and trust-based, empowering, and collaborative relationships were each 3.8 times more likely to be identified by social workers as trauma informed. Schools with a commitment to trauma awareness and training were almost 9 times more likely to be identified as trauma informed. Our results suggest that real culture and climate differences exist between schools that social workers consider trauma informed and not. However, the results also indicate that a commitment to trauma awareness and education is still one of the most significant factors in identifying a school as trauma informed.

Additionally, the entire survey instrument was able to correctly identify whether or not a school was trauma informed close to 8 out of 10 times. These findings suggest that the instrument may have value as a trauma-informed climate assessment for other schools and districts. The training and commitment index was the most successful index for identifying schools that social workers believed were trauma informed, but each climate index independently identified whether or not a school was trauma informed more than 6 out of 10 times. Although the above indices’ predictive power was better than chance, the ability of individual indices to identify trauma-informed schools may have been limited for two reasons. First, there is a very high degree of correlation between the three climate indices. Also, elements of the climate indices such as an emphasis on relationships, strengths, equity, and safety are not unique to a trauma-informed approach. For example, community schools emphasize a whole-child focus and deep connection to the surrounding community (Community Schools Forward, 2023). Physical safety, emotional safety, and supportive relationships are also commonly understood components of positive school climate (Cohen et al., 2009; Thapa et al., 2013; Wang & Degol, 2016). Although the climate indices were only moderately successful at identifying whether a school was trauma informed, they remain useful because of their ability to illustrate that social workers perceived real climate differences between schools identified as trauma informed or not trauma informed.

### Practice, Policy, and Empirical Implications

This study has implications for social welfare practice, education policy, and future empirical study of trauma-informed schools. Given the rapid growth and popularity of trauma-informed schools in the past decade and the relative nascence of their empirical study (Avery et al., 2021; Maynard et al., 2019), this article provides valuable information about measurable culture and climate differences between schools that social workers identified as trauma informed and those they did not. As our analyses show, although a commitment to trauma and the availability of trauma training were most able to properly classify a school’s type, social workers reported differences in climate perceptions on all the indices surveyed. These findings indicate that a school’s trauma awareness and focus relate to positive differences in school climate. They also point to a need to move practitioner and administrator focus beyond solely making a commitment to trauma and providing trauma training. Instead, more consideration of global practice and policy changes at the district, school, and classroom levels are needed to develop and sustain a safe, supportive, and welcoming school climate.

Given that school climate differences also related to a school’s percentage of students from historically marginalized populations, it is essential that considerations of race, poverty, and equity are included in future conceptualizations, implementations, and study of trauma-informed school climate. Prior research has shown that school staff often do not understand or prioritize race-related issues (Howard, 2020), leading some researchers to call for the centering of racial and other systemic inequities in our understanding of trauma and implementation of trauma-informed approaches in schools (Alvarez, 2020; Saleem et al., 2022). Without understanding students’ and families’ contexts and cultural heritage, it is impossible to address systemic inequities and create an inclusive environment that promotes student belonging and psychological safety (Alvarez, 2020; Astor et al., 2021; Edmondson, 2018; Zimmerman & Astor, 2021).

The intersections between trauma-informed climate, equity, belonging, and student well-being underscore the contemporary urgency of this work. A trauma-informed climate that centers equity creates conditions for authentic belonging—where students from historically marginalized backgrounds experience not only safety but also affirming recognition of their identities and cultural assets. This is critical given that experiences of racism, discrimination, and marginalization are themselves sources of trauma (Saleem et al., 2022). When schools cultivate the relational trust, psychological safety, and strengths-based approaches characteristic of trauma-informed climate, they create environments where all students can develop the secure attachments and sense of belonging fundamental to well-being and learning. Future research must consider issues of equity and capacity as they relate to trauma-informed climate.

As noted above, our analysis revealed an inverse relationship between school social worker experience and the likelihood of employment in schools identified as trauma informed. Several potential explanations for this observation warrant further investigation. Firstly, less experienced social workers may actively seek out and prioritize employment in supportive environments, a characteristic often associated with trauma-informed schools. Secondly, higher turnover rates among school social workers may be prevalent in schools with high trauma exposure. Finally, more experienced social workers might intentionally seek positions in schools with lower trauma incidence, potentially driven by factors such as burnout prevention or personal preference. We recommend future research assess how personal demographics relate to varied perceptions of school climate.

It is important to note that the climate differences we explored would not rely on the implementation of a set curriculum or program. This finding bolsters prior evidence that currently, the key differentiators between trauma-informed and non-trauma-informed schools is trauma training and supports for staff (Watson et al., 2024). Climate differences instead related to (1) the school’s commitment to creating a physically, emotionally, and psychologically safe school environment for all staff and students; (2) taking a strengths-based, whole-person lens to staff, students, and families; (3) working to create equity-centered policies and practices; and (4) promoting positive relationships between administrators and staff, among staff members, and between staff and students and their families through collaboration, empowerment, and transparency. These are commitments any school can make but they also require awareness and change at all levels of the school ecosystem. With adequate time and influence, school social workers can support and encourage these efforts as part of their everyday responsibilities of building and sustaining a positive school climate.

Beyond identifying these climate characteristics, our findings have important implications for implementation fidelity, workforce development, school improvement planning, and policy decisions. For implementation fidelity, our results indicate that schools must attend to organizational climate shifts beyond training alone—establishing metrics that assess safety, equity focus, and relationship quality, not merely professional development participation. For workforce development, the inverse relationship between social worker experience and trauma-informed school employment raises questions about recruitment, retention, and support structures in high-trauma environments. In relation to school improvement planning, our instrument provides a practical assessment tool that enables schools to integrate a commitment to trauma-informed climate into broader improvement cycles rather than treating it as an isolated initiative. Finally, these findings suggest that state and district policies should incentivize comprehensive school climate efforts through funding for sustained coaching and technical assistance, rather than compliance-focused training mandates alone.

Federal legislation typically calls upon U.S. schools to implement evidence-based mental health practices. This is also true regarding trauma (e.g., Every Student Succeeds Act, 2015). There are numerous challenges associated with scaling up EBPs. Their cost, frequent lack of cultural and contextual relevance, the difficulty of implementing and sustaining them with fidelity, and the fragmented school programming that results from this approach is not the most effective use of finite school resources (Adelman & Taylor, 2018; Osher et al., 2021). As a result, many EBPs cannot be sustained long-term (Fixsen et al., 2013). Instead of mandating specific tools for practice, policymakers must advocate that schools meet certain guidelines for student support and learning outcomes, but allow for tailored, whole-school, and district-led approaches geared to their unique context and population.

This study also provided an opportunity to pilot a new trauma-informed culture and climate instrument. Our findings indicate that differences in culture and climate between trauma-informed and non-trauma-informed schools can be identified using this new instrument, which is valuable from both a practice and empirical perspective. Schools need tools to monitor their change strategies and additional study of whole-school, trauma-informed approaches is critical because implementation has outpaced empirical support. Of course, additional work needs to be undertaken to validate this instrument.

The instrument piloted in this study offers practical applications for educational stakeholders at multiple levels. District leaders can use it to conduct baseline assessments across schools, identify system-wide patterns, guide resource allocation to schools showing lower climate scores, and track progress over time. School improvement teams can employ the instrument to conduct annual climate inventories across multiple stakeholder groups, identify priority dimensions for focused improvement, set measurable goals within those dimensions, and monitor progress through re-administration. State education agencies could incorporate the assessment into monitoring systems, use aggregated data to identify regional needs requiring policy responses, develop clear implementation guidance across all four culture and climate dimensions, and evaluate whether state trauma-informed initiatives are producing intended improvements. Critically, this instrument measures perceptions of climate—safety, equity focus, and relationship quality—rather than fidelity to specific programs. As our findings demonstrate, climate differences relate to fundamental commitments that any school can make, not adoption of branded curricula. Stakeholders should use this tool to guide ongoing improvement cycles and incorporate multiple perspectives, recognizing that authentic climate change requires sustained commitment, adequate resources, and willingness to address systemic inequities beyond assessment alone.

### Strengths, Limitations, and Future Research

There are limitations to this study that should be acknowledged. We utilized a national sample of school social workers, but it was not a representative sample. Some states and demographics were represented by only a handful of individuals. As a result, comparisons were not possible by state and some racial and gender identities had to be combined in our analyses. In addition, only school social workers were surveyed, but best practices in school climate research would involve inclusion of all staff and students in the school environment to get a more complete climate picture. Due to the utilization of a national sample instead of undertaking this study in one or a few schools, it is unclear whether differences in experiences by gender and racial/ethnic identity we found in preliminary analyses were due to staffing differences between trauma-informed and non-trauma-informed schools. Study of climate perceptions across multiple gender and racial/ethnic identities in one school would enable better assessment of how trauma-informed climate may differ by demographic. This study piloted a new trauma-informed culture and climate instrument because adequate alternatives did not exist. Additional work is needed to validate the instrument.

Trauma-informed approaches in schools have been readily implemented in the last couple of decades but empirical study of these approaches remains limited. Due to a lingering dearth of empirical support for whole-school, trauma-informed approaches (Watson & Astor, 2025; Avery et al., 2021; Gherardi et al., 2020; Maynard et al., 2019), significant additional research is suggested. Future research should include intentional assessment of differences in perceptions of trauma-informed culture and climate in one school or district based on respondent role (e.g., students, certificated and classified staff, etc.) and identity (e.g., race, gender, ethnicity, LGBTQ+ status). Although both organizational and school researchers tend to distribute surveys to only one constituent group (Berkowitz et al., 2017; Ostroff et al., 2013; Schneider et al., 2013), the viewpoints of multiple stakeholders enrich understanding of multidimensional constructs like trauma-informed culture and climate and should be the goal of organizational assessment. Additional research is also needed to understand what differences in schools are intentional and what is currently being done by schools in the name of being trauma informed. Very few school climate studies have utilized qualitative methods. This is an oversight because qualitative methods, including focus groups, interviews, and observations, can better explore processes and aspects of climate that are more neutral instead of purely positive or negative (Wang & Degol, 2016). Multiple case studies of trauma-informed schools that include participant observation, review of disciplinary and attendance data, and changed policies would be beneficial.

This article did not present findings on differences in individual experiences of school culture or climate based on race, gender, or other personal demographics. However, prior research has shown that social identity impacts individuals’ experiences of an organization’s policies, practices, culture, and climate, including in schools (Chung, 1997; Hogg & Terry, 2014; Thapa et al., 2013). Considerations of race, equity, diversity, and inclusion should be included in future conceptualizations and assessments of trauma-informed or healing-centered schools. Without these additions, we will never be able to adequately understand or study supportive and healing school spaces. Such study also needs to consider how structural inequities in terms of funding, staffing, and other resources may affect school climate. Would equal funding across all schools and districts improve child–-staff ratios to an extent that a focus on “trauma-informed” or “healing-centered” approaches was no longer necessary? Over the longer term, researchers also must explore staff and student outcomes related to trauma-informed culture and climate. It is important to understand whether differences in trauma-informed culture and climate result in improved academic and behavioral outcomes for students, higher staff retention, or other benefits.
